# Betulinic acid-mediating miRNA-365 inhibited the progression of pancreatic cancer

**DOI:** 10.32604/or.2023.026959

**Published:** 2023-06-27

**Authors:** XIN LI, WENKAI JIANG, WANCHENG LI, SHI DONG, YAN DU, HUI ZHANG, WENCE ZHOU

**Affiliations:** 1 Department of General Surgery, The Second Hospital of Lanzhou University, Lanzhou, 730000, China; 2 The First Clinical Medical College, Lanzhou University, Lanzhou, 730000, China; 3 The Second Clinical Medical College, Lanzhou University, Lanzhou, 730000, China

**Keywords:** Betulinic acid, Pancreatic cancer, miRNA-365, BTG2, IL-6

## Abstract

**Background:**

The dilemma of pancreatic cancer treatment has become a global challenge. For this reason, effective, feasible, and new medical methods are currently much-needed. Betulinic acid (BA) has been valued as a potential therapy for pancreatic cancer. However, the mechanism by which BA exerts an inhibitory effect on the development of pancreatic cancer remains elusive.

**Methods:**

A rat model and two cell models of pancreatic cancer were established, and the effect of BA on pancreatic cancer was verified *in vivo* and *in vitro* by using MTT, Transwell, flow cytometry, RT-PCR, Elisa and immunohistochemistry. At the same time, miR-365 inhibitors were introduced to test whether BA played a role in mediating miR-365.

**Results:**

BA can significantly inhibit the proliferation and invasion of pancreatic cancer cells and promote apoptosis. *In vivo* experiments, BA can significantly lower the number of cancer cells and tumor volume in the rat model of pancreatic cancer. *In vitro*, it was found that BA inhibited the protein level and phosphorylation level of AKT/STAT3 by mediating the expression of miR365/BTG2/IL-6. Like BA, miR-365 inhibitors also significantly inhibited cell viability and invasion ability, and inhibited the protein level and phosphorylation level of AKT/STAT3 by changing the expression of BTG2/IL-6, and their combination had a synergistic effect.

**Conclusion:**

BA inhibits AKT/STAT3 expression and phosphorylation by modulating miR-365/BTG2/IL-6 expression, and BA inhibits the progression of pancreatic cancer through the aforementioned mechanism.

## Introduction

Pancreatic cancer is a lethal disease in digestive system. The most recent data of the American Cancer Society show that the prognosis of those with advanced pancreatic cancer is extremely poor, with a five-year survival rate of only 12% [1]. With resectable surgery as the first option, the chance of curing pancreatic cancer is slim, and it is possible to recur after surgery [2]. Despite significant advancements in recent years in tumor chemotherapy, radiotherapy, immunotherapy, targeted therapy, and combination therapy, patients with pancreatic cancer can only benefit from these treatments to a limited extent [3,4]. Given this, the situation of the treatment of pancreatic cancer is still grim. On this premise, some of the current studies focus on the development of novel anti-tumor drugs. Natural substances are among them, and because of their numerous biological activities and mild side effects, they have emerged as one of the key sources of research on anti-tumor drugs [5].

Betulinic acid (BA), a pentacyclic triterpenoid compound, was found in birch bark and thorny jujube seeds. It has a variety of biological activities [6]. Nowadays, BA is widely used to investigate its antitumor effect due to its few toxic side effects. Studies have shown that BA can trigger apoptosis of cancer cells by blocking the cell cycle [7], changing the mitochondrial membrane potential [8] and inhibiting the stemness of CSCs and EMT [9]. However, it remains unknown how BA affects the onset and progression of pancreatic cancer, as well as the related mechanism. Therefore, research on the role of BA in pancreatic cancer may be important for developing novel anti-tumor strategies.

miRNA is a small class of endogenous single-stranded RNA that typically serves as a negative regulator by targeting specific miRNA for the inhibition of degradation or translation [10]. The important role of miRNA in drug resistance of pancreatic cancer has been demonstrated [11]. Some studies have suggested that the combined miRNA regulation therapy was a feasible and effective way to treat pancreatic cancer [12]. Yet, it is still unclear how BA affects miRNA in pancreatic cancer. Based on this, we carried out animal studies and discovered that the malignant degree of the tumor declined and miRNA-365 was blocked in the BA-treated mice model of pancreatic cancer. According to our previous study, miR-365 can inhibit the expression of BTG2, thereby activating the FAK/AKT pathway and promoting the development of PDAC. Inhibition of miR-365 down-regulated the proliferation, migration and invasion potential of PDAC cells [13]. Therefore, the main purpose of this study was to verify the effect of BA on miR-365 and its possible mechanism in pancreatic cancer.

## Materials and Methods

### Animals and model establishment

50 SD rats with a body weight of (200 ± 20) g were purchased from Lanzhou Veterinary Research Institute, Chinese Academy of Agricultural Sciences. After free feeding for one week, they were used to build a model of pancreatic cancer. The models, mainly based on previous research, were also known as DMBA induction method [14–16]. The methods were as follows: the rats were anesthetized using pentobarbital sodium, and their abdomen was depilated and disinfected. A 1–2 cm incision was made in the abdomen to expose the pancreas. DMBA (9 mg) was implanted between pancreatic tissues and parenchyma, and then the pancreatic parenchyma and abdominal incision were sutured in order to prevent infection. 4 weeks later, the rats were sacrificed, and tissues were collected to determine whether the model was successfully built.

The model rats were randomly divided into 4 groups and named as model group (Model), BA (Sigma-Aldrich) low-dose group (Low, 50 mg/kg), medium-dose group (Medium, 100 mg/kg) and high-dose group (High, 150 mg/kg), respectively. The rats were euthanized 15 days after administration, and the samples were collected for subsequent analysis.

The rats in the blank control group only had their wounds heal and did not undergo DMBA transplantation. All animal experiments were carried out under the supervision and guidance of the Animal Ethics Committee of Lanzhou University and the First Affiliated Hospital. All animals were handled according to the animal welfare guidelines and approved by the Ethics Committee of the First Hospital of Lanzhou University (LDYYLL2021.09).

### Histopathological examination

After the frozen sections were fixed and sealed, Akt and STAT3 specific primary antibodies were added and incubated overnight at 4°C. Subsequently, the biotinylated secondary antibody was incubated at 37°C for 30 min. Streptomycin-labeled HRP was added and incubated for 30 min. Finally, the slices were colored and counterstained with DAB, sealed with neutral gum, and photographed under a microscope for observation and analysis.

### H&E

Paraffin-embedded tissue sections were deparaffinized and hydrated, the sample was stained with hematoxylin and let stand at room temperature for 5 min. Eosin was added after washing, and the sample was then incubated for about 2 min at room temperature. After being rinsed with gradient ethanol, a neutral gel was then used for sealing. The sections were observed and photographed under a microscope. 3 experienced pathologists analyzed the results.

### ELISA

IL-6 assay was performed according to the instructions of the manufacturer of IL-6 kit (Beyotime, PI328). The serum to be tested and the standards with different concentrations were added to the corresponding wells and incubated for 2 h at 37°C. Biotinylated antibody and HRP-Streptavidin were then added and incubated, respectively, and TMB was added to colorate. A450 absorbance was measured.

### Cell culture

PANC-1 cells were derived from ATCC. The cells were cultured in DMEM medium supplemented with 10% FBS and 1% penicillom-ycin. BxPC-3 cells were derived from the Cell Bank of the Chinese Academy of Sciences. The cells were cultured in RPMI-1640 medium supplemented with 10% FBS and 1% penicillom-ycin. The cells were cultured in an incubator with 5% CO_2_ at 37°C.

### miR-365 inhibitor

miR-365 inhibitor was purchased from Shanghai GenePharma. Lipofectamine 3000 was adopted for transfection experiment. The cells were subcultured in a 6-well plate and cultured for 24 h. The inhibitor was mixed with the transfection reagent, placed at 37°C for 15 min, and then added to cells. After 48 h, the cells were collected and the expression level of miR-365 was tested by RT-PCR.

### MTT assay

The cell viability was determined by MTT assay. The cells were subcultured in a 96-well plate and cultured for 24 h for different treatments. MTT solution was added and incubated for 4 h, and then DMSO was added to terminate the reaction. The absorbance was measured at 450 nm under a microplate reader.

The cells were treated with 0, 10, 20 and 30 µM BA [2,15], and the treatment lasted 24, 48 and 72 h, respectively. Each treatment was repeated for 6 times, and the concentration of BA IC50 was screened for subsequent experiment. The experiment was divided into Control group, Inhibitor-NC (miR-365 inhibitor NC) group, Inhibitor group (miR-365 inhibitor), BA group (IC50 concentration) and BA+ Inhibitor group. The cells were subjected to different treatments and the cell viability was determined 48 h later.

### Transwell invasion assay

The invasion assay was performed in a Transwell chamber, with the upper surface of the 8-μm (pore size) membrane being coated with Matrigel. The cells undergoing different treatments were inoculated in a Transwell chamber, and a complete medium with 10% FBS was added to the outer cavity of chamber. After incubating at 37°C for 24 h, the chamber was removed and cleaned with PBS. After that, 4% paraformaldehyde was fixed at 37°C for 30 min, and crystal violet staining was performed. After excessive cells in the upper layer of chamber were removed, the number of migrated cells was observed under a microscope. 3 fields were randomly selected as replicates in each sample.

### Flow cytometry

The cells were inoculated in a 6-well plate. After the adhesion and fusion of cells, different concentrations of BA (0, 10, 20, 30 µM) were added, and the cells were collected for 48 h. Annexin V-FITC was added to the suspended cells and incubated for 15 min, followed by PI for 5 min. FACS C6 was adopted to detect cell apoptosis and FlowJo was used for data analysis.

### Western blotting

The cells undergoing different treatments were centrifuged and collected, supplemented with RIPA lysate, and placed on ice for 30 min to extract total protein. The proteins were separated by SDS-PAGE and transferred to an activated PVDF membrane.

After blocking with 5%BSA, specific primary antibodies were added, and incubated overnight at 4°C. After washing, HRP-labeled secondary antibodies were added and incubated at 37°C for 1 h. The target protein was colorated with ECL method, and the images obtained by the gel imaging system were analyzed semi-quantitatively with Image J software. The antibodies used in the experiment were BTG2 (Abcam, AB244260), IL-6 (Abcam, AB233706), p-AKT (CST, 4060S), AKT (CST, 4685S), p-STAT3 (CST, 9145S) and STAT3 (CST, 9139S), respectively.

### qRT-PCR

Total RNA was extracted from the samples with Trizol. After determining the concentration and quality of RNA, cDNA was synthesized according to the instructions of reverse transcription kit (QIAGEN, 205411), TaKaRa’s commercial instructions. Taking 2 µL cDNA product as the template, the relative expression levels of target genes were analyzed by QuantiNova SYBR Green PCR Kit (QIAGEN, 208054). The primer sequences are shown in Table 1.

**Table 1:** Sequences of target genes

Genes	Forward	Reverse
miRNA365 (human)	TAATGCCCCTAAAAATCCTTAT	GCGAGCACAGAATTAATACGAC
miRNA365 (rat)	CGCGTAATGCCCCTAAAAAT	AGTGCAGGGTCCGAGGTATT
BTG2 (rat)	CAACCACAAGATGGACCCCA	GATGCGGTAGGACACTTCGT
BTG2	TAACGCTGTCTTGTGGACCC	TTAAGCCTCTGCTCGCTCAC
IL-6	GGCACTGGCAGAAAACAACC	GCAAGTCTCCTCATTGAATCC
AKT	CACACCACCTGACCAAGATG	CCTCAGAGACACGGCCTTAG
STAT3	CAGCAGCTTGACACACGGTA	AAACACCAAAGTGGCATGTGA
U6	CTCGCTTCGGCAGCACA	AACGCTTCACGAATTTGCGT
ß-actin	CTGAGAGGGAAATCGTGCGT	CCACAGGATTCCATACCCAAGA

miRNA-specific TaqMan miRNA assay Kit (Thermo Fisher Scientific Waltham, MA, USA) was used to analyze the expression level of miRNA. U6 was used for gene normalization. The relative expression value was expressed by the value of 2^−∆∆Ct^.

### Statistical analysis

SPSS 20.0 and GraphPad Prism 7.0 were adopted for statistical analysis and figure rendering. All data were expressed as Mean ± SD. The comparison between multiple groups was done with ANOVA and the comparison between two groups was done with *t*-test. *p* < 0.05 indicated the difference was statistically significant.

## Results

### BA inhibited the proliferation and invasion of pancreatic cancer and induced cell apoptosis

**Figure 1:**
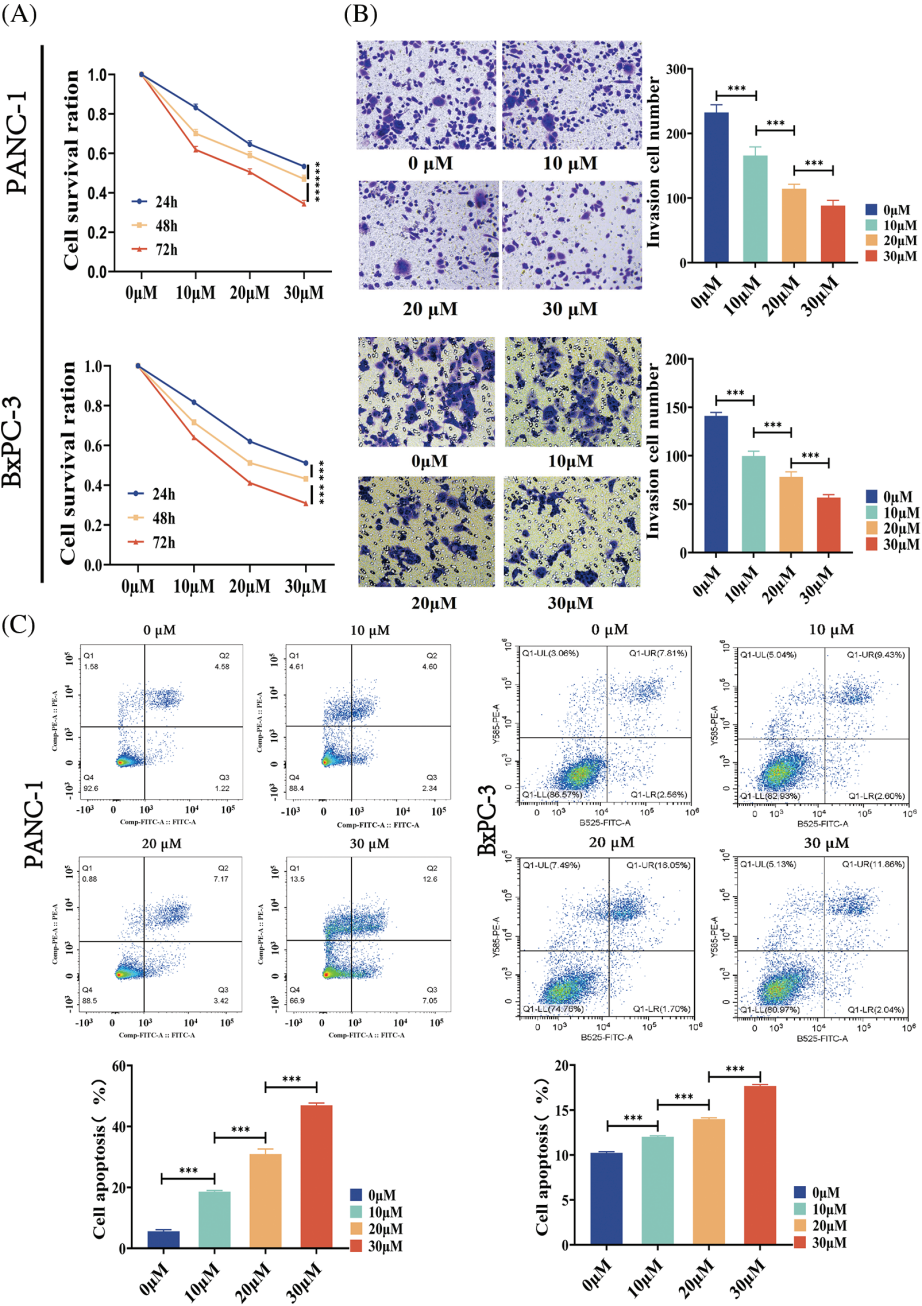
BA inhibited the cell invasion and promoted the cell apoptosis (A) MTT determined the effect of BA on the proliferation of PANC-1 and BXPC-3 cells;(B) Transwell experiments were used to determine how different concentrations of BA affected the migration capacity of PANC-1 and BxPC-3 cells.; (C) Effect of BA on apoptosis of PANC-1 and BxPC-3 cells measured by flow cytometry. * *p* < 0.05; ** *p* < 0.01; *** *p* < 0.001.

We investigated the effect of BA on the biological behavior of pancreatic cancer cells, so as to better understand the role of BA in pancreatic cancer. PANC-1 and BXPC-3 cells were selected as pancreatic cancer cell lines. First of all, the MTT assay was used to assess changes in the proliferation ability of pancreatic cancer cells after BA treatment. BA had a concentration-dependent inhibitory effect on the proliferation of pancreatic cancer cells, as shown in Fig. 1A, and with the increase of concentration, cell proliferation was inhibited at different time points. Transwell experiments were then carried out to examine the changes in the migration ability of pancreatic cancer cells after BA treatment. As shown in Fig. 1B, as the concentration of BA increased, pancreatic cancer cells’ ability to migrate weakened. Finally, flow cytometry was adopted to investigate the effect of BA on the apoptosis of pancreatic cancer cells. The degree of apoptosis of pancreatic cancer cells increased with the increase of the concentration of BA, as shown in Fig. 1C.

### BA alleviated the progression of pancreatic cancer via the regulation on miR-365/BTG2 and IL-6/AKT/STAT3 in vivo

**Figure 2:**
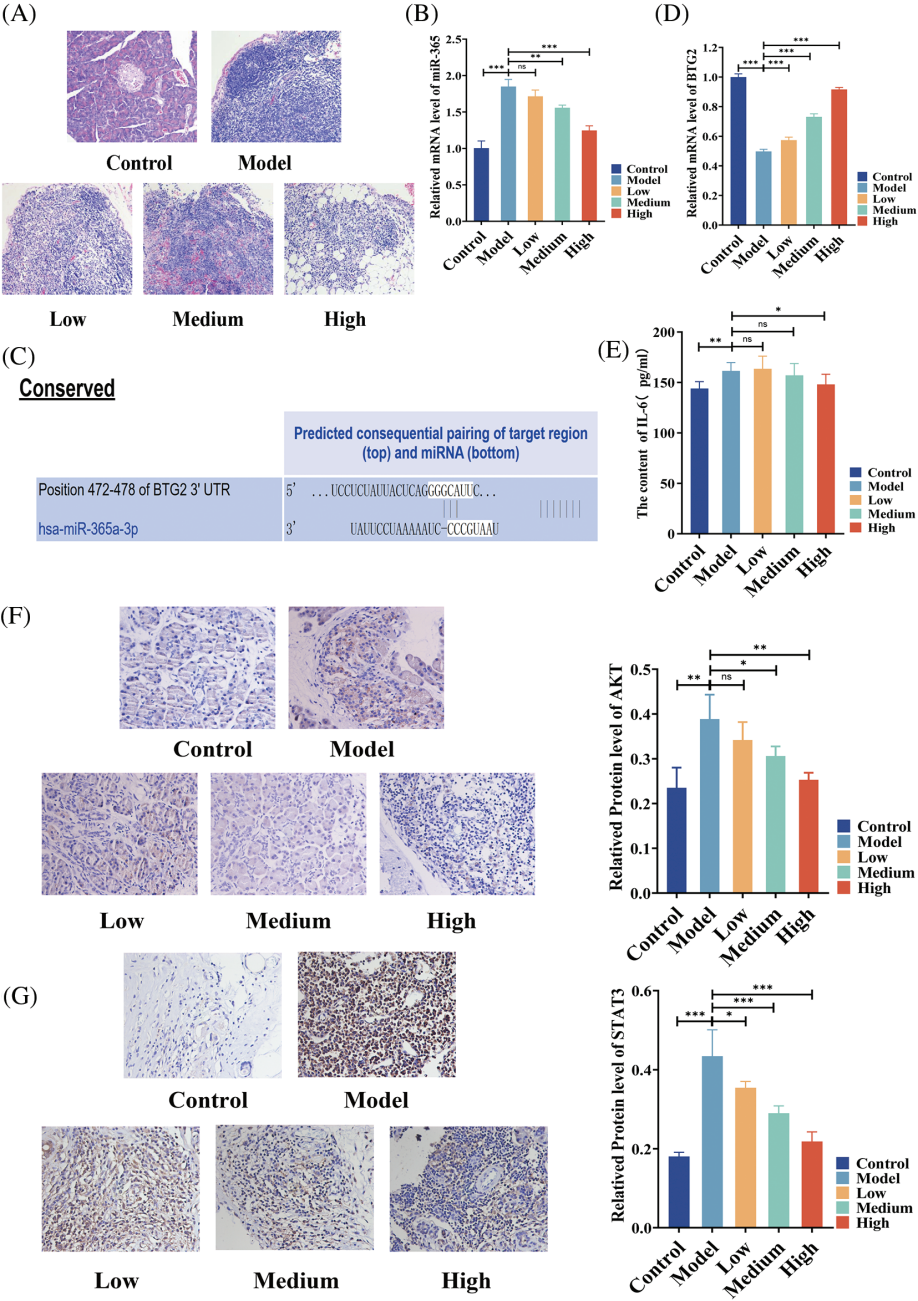
Expressions of miR365, IL-6, AKT and STAT3 were downregulated by BA *in vivo* (A) H&E pathological section of pancreatic cancer (200X); (B) The level of miR365 was reduced by BA; (C) The relationship between miR-365 and BTG2 was analyzed using the Targetscan database; (D) Determination of mRNA level of BTG2 in tumor tissue; (E) BA inhibited the protein level of IL-6; (F) Expression of AKT were determined by immunohistochemistry (400X); (G) Expression of STAT3 were determined by immunohistochemistry (400X). * *p* < 0.05; ** *p* < 0.01; *** *p* < 0.001.

In an effort to study the mechanism of the effect of BA on pancreatic cancer, a rat model of pancreatic cancer was built and BA with different concentrations, that is, low, medium and high, was used. H&E analysis showed that in the model group, the tumor cells were densely arranged, inflammatory cells were infiltrated, and vascular hyperplasia occurred. After BA treatment, the number of cancer cells decreased, which was negatively correlated with the dose of BA (Fig. 2A). Following that, we discovered that miR-365 was overexpressed in the pancreatic cancer model group, compared with the control group. BA significantly inhibited the level of miR-365 in a dose-dependent manner when compared with the model group (Fig. 2B). Then through the Targetscan database, we discovered that miR-365a-3p and BTG2 had binding sites (Fig. 2C). On this basis, we evaluated the changes in the level of BTG2 mRNA in the BA-mediated pancreatic cancer animal model. The expression of BTG2 mRNA in the model group was lower than that in the blank group, and BA can up-regulate the level of BTG2 mRNA in tumor tissues, compared with the model group (Fig. 2D). Subsequently, the model group and the BA group were compared in terms of the expression levels of IL-6, AKT, and STAT3. As expected, the secretion level of IL-6 was significantly reduced in the BA high level group, and the immunohistochemical results indicated that the proteins of AKT and STAT3 were inhibited by BA (Figs. 2E and 2F). In this regard, we preliminarily speculated that BA may inhibit the expression of IL-6/AKT/STAT3 by mediating the expression of miR-365/BTG2.

### BA inhibited the proliferation and invasion of pancreatic cancer cells by targeting miR-365

**Figure 3:**
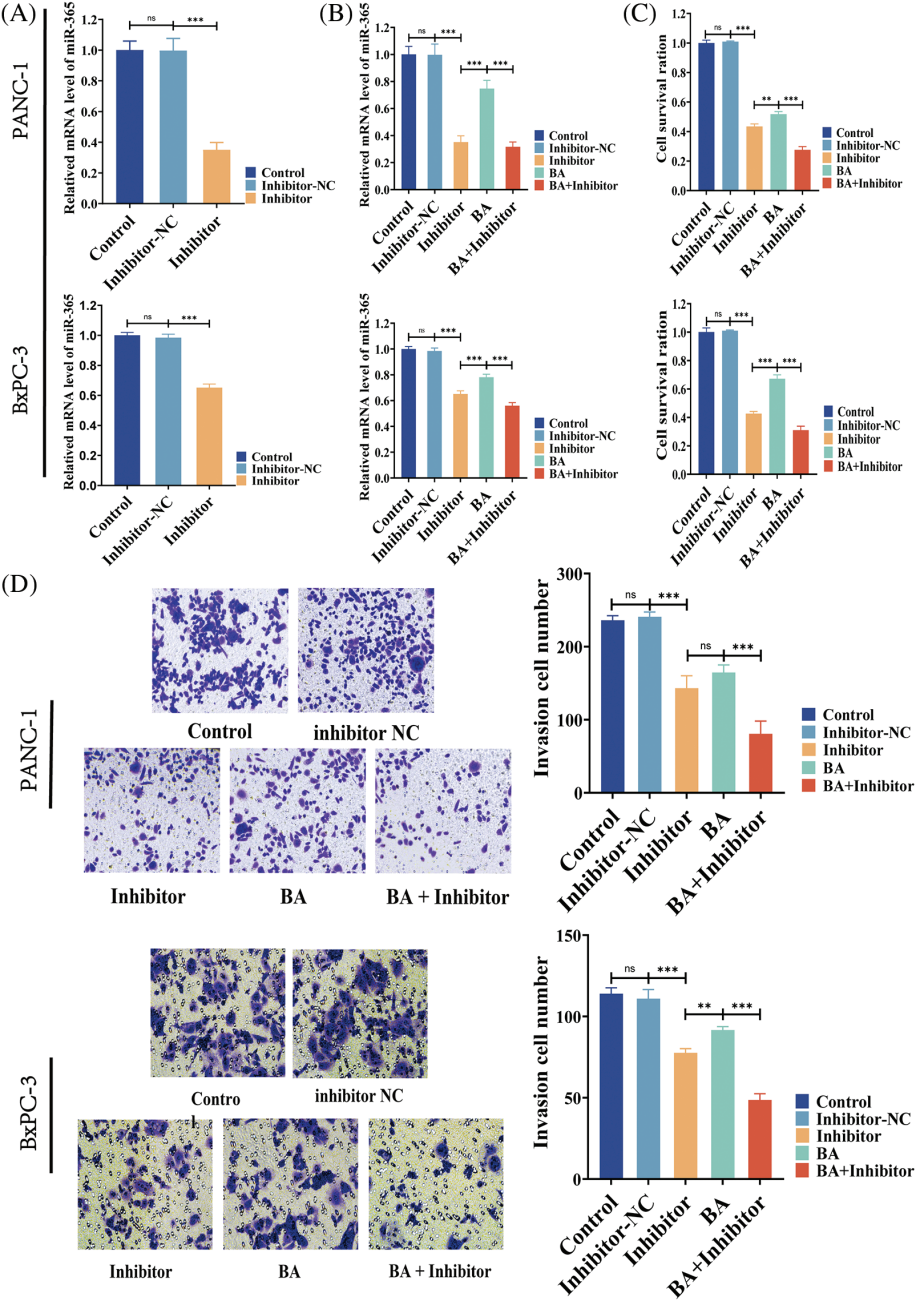
BA mediating to miR365 alleviated pancreatic cancer cells proliferation and invasion (A) Effect of miR365 inhibitor in PANC-1 and BxPC-3 cells; (B) The effects of BA and miR365 inhibitors on miR-365 expression in PANC-1 and BxPC-3 cells were determined using RT-PCR; (C) Effect of BA on proliferation of PANC-1 and BxPC-3 cells by MTT assay; (D) Effect of BA on invasion of PANC-1 and BxPC-3 cells by Transwell. * *p* < 0.05; ** *p* < 0.01; *** *p* < 0.001.

According to two previous cell MTT experiments, the 48-h IC50 of BA in PANC-1 was 27.57 and 22.47 μM in BxPC-3. Therefore, the following experiment selected the condition that BA was 28 μM in PANC-1 and 23 μM in BxPC-3. To investigate whether BA played a role in pancreatic cancer by mediating miR-365, we introduced the inhibitor of miR-365 into the experiment to verify whether the regulatory effect of BA on miR-365 was similar to that of the inhibitors. As shown in the figure, the inhibitor significantly down-regulated the level of miR-365, which was consistent with the effect of BA (Figs. 3A and 3B). At the same time, both BA and inhibitor had an inhibitory effect on the proliferation and invasion ability of pancreatic cancer cells, and the combined effect of the two further weakened the proliferation and invasion ability of cancer cells (Figs. 3C and 3D).

### BA suppressed AKT/STAT3 expression by modulating miR-365/BTG2/IL-6

**Figure 4:**
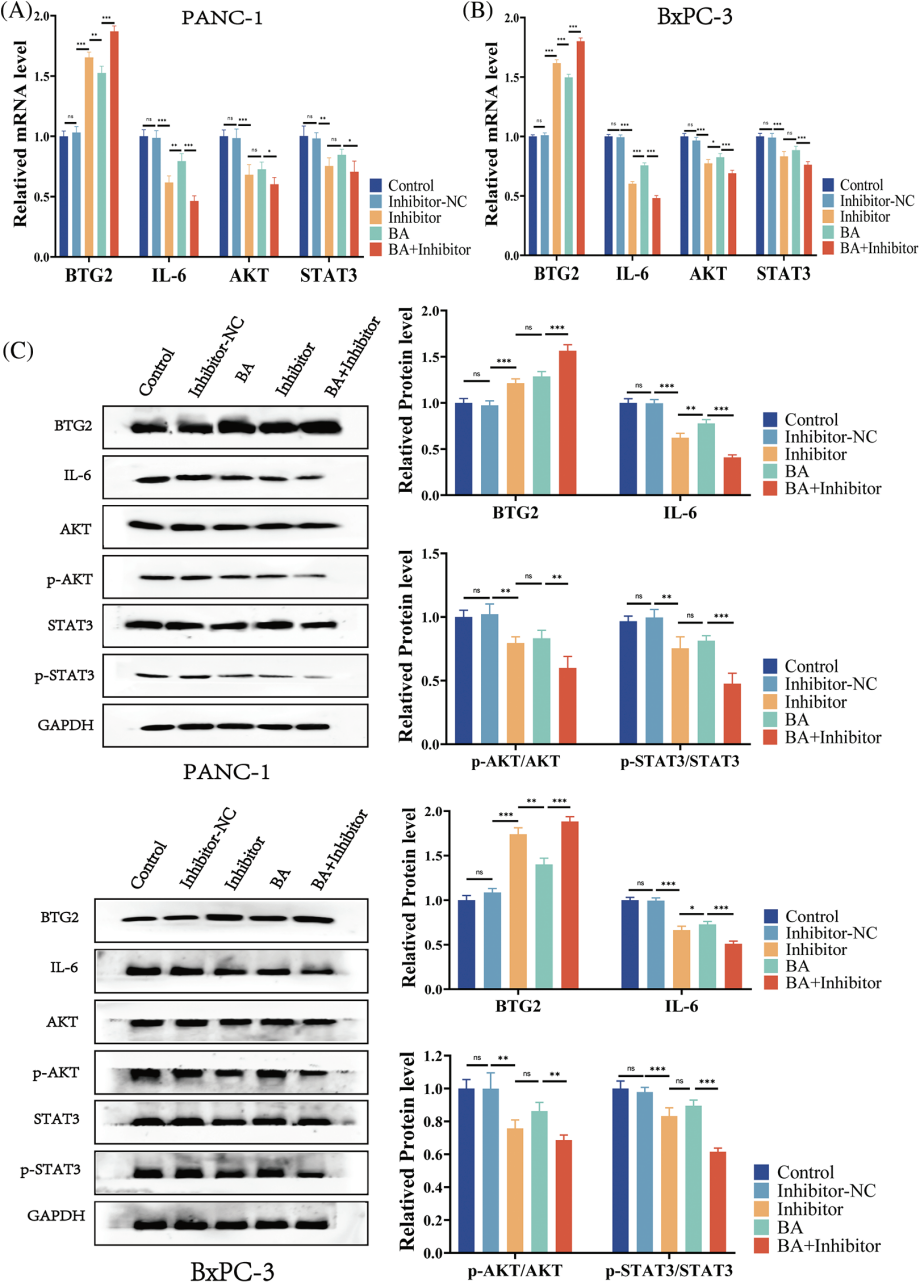
BA inhibits miR-365 expression, causing changes in BTG2/BTG2 and thus inhibiting IL-6/AKT/STAT3 expression (A) The expression of BTG2/IL-6 is measured using QT-PCR in two cell strains treated with BA and miR-365 inhibitors; (B) The expression of AKT/STAT3 is measured using QT-PCR in two cell strains treated with BA and miR-365 inhibitors; (C) WB quantified the changes in BTG2/IL-6/STAT3/P-STAT3/AKT/P-AKT expression in two pancreatic cancer cells treated with BA and miR-365 inhibitor. * *p* < 0.05; ** *p* < 0.01; *** *p* < 0.001.

To further investigate the mechanism of the inhibition of progression of pancreatic cancer by miR-365/BTG2 mediated by BA, we first measured the expression of BA-mediated miR-365/BTG2 in two pancreatic cancer cell lines. We discovered that both BA and miR-365 inhibitors can promote BTG2 expression (Figs. 4A–4C), which was consistent with previous studies. Following that, we measured IL-6/AKT/STAT3 expression and discovered that BA and miR-365 inhibitors can inhibit IL-6/AKT/STAT3 expression and AKT/STAT3 phosphorylation (Figs. 4A–4C). We concluded from the above experimental results that BA ultimately inhibited progression of pancreatic cancer by mediating miR-365/BTG2 and then inhibiting IL-6/AKT/STAT3 expression.

## Discussion

Pancreatic cancer is one of the most deadly digestive tract tumors, whose morbidity and mortality increase rapidly on the global context. Current therapies for patients with advanced pancreatic cancer are ineffective, due to drug resistance in chemotherapy, low responsiveness to immunotherapy and targeted therapy, and other factors [17,18]. Consequently, one of the research hotspots is the development of safe and effective new drugs to inhibit the progression of pancreatic cancer. BA, a natural compound, has received increasing attention in recent years because of its potent and diverse anti-tumor properties, but its anti-tumor effect and potential mechanism in pancreatic cancer remain unknown [19]. As a result, we investigated the effect and mechanism of BA in pancreatic cancer *in vivo* and *in vitro* in this study. BA inhibits the proliferation, migration, and invasion of pancreatic cancer cells *in vivo* and *in vitro* through inhibiting IL-6 protein expression and AKT/STAT3 protein phosphorylation by mediating miR-365/BTG2 axis. Therefore, BA may be a potential therapy for pancreatic cancer.

Previous studies have shown that combining BA and mitramycin A can inhibit the angiogenesis, proliferation and invasion of pancreatic cancer by down-regulating SP1 [20]. To that end, we first tested the effect of BA on the biological behavior of pancreatic cancer cells. The findings suggested that BA can inhibit the proliferation, apoptosis, and invasion of pancreatic cancer cells, which was consistent with previous findings indicating that BA could inhibit the progression of pancreatic cancer.

MicroRNA is a small non-coding RNA that is regulated after transcription by complementary pairing [21]. Although miRNA is encoded by only about 3% of human genes, it can influence about 30% of human protein coding genes [22]. It can control a variety of cancer and development processes because of the broad range of targets it has. Some studies have confirmed that miR-365 in exocrine derived from tumor-associated macrophages can induce resistance to gemcitabine in PDAC mice, and that miR-365 inhibition can promote gemcitabine sensitivity [11]. Moreover, several studies have demonstrated that miR-365 can enhance tumors by up-regulating molecules such as S100P and DNA binding 2 [23]. On this basis, we examined the changes in miR-365 expression in pancreatic cancer cells induced by BA and discovered that miR-365 expression was down-regulated in two pancreatic cancer cell lines. Combined with the above experimental findings, it was found that BA can have an anti-tumor effect by inhibiting miR-365.

The main functions of BTG2 are to control the cycle, apoptosis, and differentiation of cells [24]. Its connection to tumor progression is being slowly revealed. Several findings have shown that BTG2 has a tumor-inhibiting impact [25]. In a previous study, we discovered an interaction between BTG2 and miR-365 through dual-luciferase reporting experiment, and we then discovered that M2-type macrophages inhibited BTG2 expression by secreting extracellular vesicles containing miR-365, activating the FAK/AKT pathway and promoting the development of PDAC [13]. In addition, it has been reported that BTG2 can reduce STAT3 phosphorylation, which in turn lowers IL-6 production in fibroblasts [26]. On this basis, we further investigated the changes in miR-365/BTG2/IL-6 caused by BA and discovered that both BA and miR-365 inhibitors could increase the expression of BTG2 and reduce the level of IL-6 in pancreatic cancer cells, and the combination of BA and miR-365 inhibitors could result in better outcome.

IL-6 is a key immune response factor, and the activity of pancreatic cancer cells can be stimulated by IL-6, which can also encourage cell growth, EMT, and immune evasion of tumor cells [27,28]. Furthermore, IL-6 can cause synergistic amplification with other cytokines to facilitate progression of pancreatic cancer. Thus, targeting IL-6 may be a promising treatment option for pancreatic cancer [27]. Moreover, the dysregulation of IL-6’s downstream signals also plays an important role in the occurrence and development of pancreatic cancer [29]. STAT3 is a cytoplasmic transcription factor that has been found to be overactivated in many tumors and is associated with a poor prognosis [30]. STAT3 not only directly regulates the target gene to promote the progression of pancreatic cancer, but also is associated with the formation of immunosuppressive microenvironment and the therapeutic resistance of pancreatic cancer [31]. The activation state of IL-6/STAT3, as a key signal pathway, and the activated Kras can enhance the development of PDAC mice model [32]. To contribute to the development of an immunosuppressive microenvironment in pancreatic cancer, the IL-6/STAT3 axis can further encourage the growth of MDSCs and other immunosuppressive cells or increase the number of Treg cells [33]. Recent studies have shown that Raloxifene inhibits pancreatic cancer *in vivo* and *in vitro* via IL-6/gp130/STAT3 signaling [34]. What’s more, it has also been revealed that IL-6/AKT signal is inappropriately activated in several malignancies, including pancreatic cancer and gastric cancer, and its activation can enhance tumor development and resistance to some chemotherapeutic medicines [35]. Studies have found that the IL-6/AKT signaling pathway promotes Mcl-1 expression in cholangiocarcinoma, which in turn mediates tumor necrosis factor-related apoptosis-inducing ligand (TRAIL) resistance in cholangiocarcinoma patients [36]. As a result, the survival, proliferation, and glycolysis of pancreatic cancer cells can be dramatically decreased by inhibiting IL-6 and its related pathways, and the treatment resistance of pancreatic cancer cells can even be reduced [37]. Based on these findings, we further investigated how BA affected the level of downstream IL-6/STAT3/AKT by mediating miR-365/BTG2. We found that BA could reduce the expression of IL-6 and the ratio of p-STAT3/STAT3 and p-AKT/AKT by mediating miR-365/BTG2, which may be one of the mechanisms of BA in anti-pancreatic cancer.

## Conclusion

To sum up, we discover that BA inhibits the expression of IL-6 and phosphorylation of AKT/STAT3 by mediating changes in miR-365/BTG2, thereby inhibiting the progression of pancreatic cancer. These findings lend strong support to the therapeutic effect of BA in pancreatic cancer, and BA may be a promising therapy for cancer.

## Data Availability

All data needed to evaluate the conclusions in the paper are present in the paper and/or the Supplementary Materials. Additional data related to this paper may be requested from the authors.

## List of Abbrevations

ANOVA: One-way analusis of variance
AKT: Protein kinase B
BA: Betulinic acid
BAX: Bcl-2 associated x protein
BTG2: B cell translocation gene 2
CO_2_: Carbon dioxide
DMBA: 2,2′-Bis(hydroxymethyl)butyric acid
DMEM: Dulbecco’s modified eagle’s medium
EMT: Epithelial mesenchymal transition
H&E: Hematoxy lin-eosin
IC50: Half maximal inhibitory concentration
IHC: Immunohistochemistry
IL-6: Interleukin-6
MTT: 3-(4,5-dimethylthiazol-2-yl)-2,5-diphenyltetrazolium bromide
PDAC: Pancreatic ductal adenocarcinoma
RT-PCR: Real time polymerase chain reaction
STAT3: Signal transducer and activator of transcription
